# New Genomic Techniques applied to food cultures: a powerful contribution to innovative, safe, and sustainable food products

**DOI:** 10.1093/femsle/fnae010

**Published:** 2024-02-06

**Authors:** Fabio Dal Bello, Laetitia Bocquet, Audrey Bru, Svend Laulund, Ronnie Machielsen, Matteo Raneri, Vincent Sewalt, Noël van Peij, Patrice Ville, Federica Volonté, Yolanda White, Jakub Rusek

**Affiliations:** Sacco, via Manzoni 29/A, 22071 Cadorago, Italy; Lesaffre, 101 rue de Menin, 59706 Marcq-en-Baroeul, France; Lallemand SAS, 19 rue des Briquetiers, 31700 Blagnac, France; Novonesis, Gammel Venlighedsvej 14, 2970 Hoersholm, Denmark; Novonesis, Gammel Venlighedsvej 14, 2970 Hoersholm, Denmark; Sacco, via Manzoni 29/A, 22071 Cadorago, Italy; IFF, 925 Page Mill Road, Palo Alto, CA 94304, United States; DSM-Firmenich, Alexander Fleminglaan 1, 2613 AX, Delft, the Netherlands; Lesaffre, 101 rue de Menin, 59706 Marcq-en-Baroeul, France; Sacco, via Manzoni 29/A, 22071 Cadorago, Italy; Lallemand SAS, 19 rue des Briquetiers, 31700 Blagnac, France; EFFCA - European Food and Fermentation Cultures Association, c/o Kellen, 188 Avenue de Tervueren, Brussels, Postbox 4, 1150 Brussels, Belgium

**Keywords:** New Genomic Techniques, genome editing, GM regulatory framework, sustainability, nontransgenic, food cultures

## Abstract

Nontransgenic New Genomic Techniques (NGTs) have emerged as a promising tool for food industries, allowing food cultures to contribute to an innovative, safe, and more sustainable food system. NGTs have the potential to be applied to microorganisms, delivering on challenging performance traits like texture, flavour, and an increase of nutritional value. This paper brings insights on how nontransgenic NGTs applied to food cultures could be beneficial to the sector, enabling food industries to generate innovative, safe, and sustainable products for European consumers. Microorganisms derived from NGTs have the potentials of becoming an important contribution to achieve the ambitious targets set by the European ‘Green Deal’ and ‘Farm to Fork’ policies. To encourage the development of NGT-derived microorganisms, the current EU regulatory framework should be adapted. These technologies allow the introduction of a precise, minimal DNA modification in microbial genomes resulting in optimized products carrying features that could also be achieved by spontaneous natural genetic evolution. The possibility to use NGTs as a tool to improve food safety, sustainability, and quality is the bottleneck in food culture developments, as it currently relies on lengthy natural evolution strategies or on untargeted random mutagenesis.

## Teaser

Nontransgenic New Genomic Techniques offer a promising path for the food industry to create innovative, safe, and sustainable microbial products, but EU regulations need adjustment to fully harness their potential.

## Introduction

The European ‘Green Deal’ and ‘Farm to Fork’ policies aim to make food systems more sustainable, contributing to carbon neutrality and circular economy. This involves significant advancements in agricultural and food production to address climate change, sustainable production demands, decarbonization, and reducing food waste. The increasing market requirements for food diversity, safety, and sustainability are driving the food industry to innovate rapidly, especially in developing plant-based alternatives like dairy and meat substitutes, which must meet high standards in texture, flavour, and nutritional value (Elhalis et al. 2023).

Food cultures as described in this paper are used in food processing and/or as food ingredients, as defined in the Article 2 of Regulation (EC) No 1169/2011 (e.g. dairy products, meats, beverages, breads, vegetables and other plant-based products). They contribute to several food properties, such as flavour, texture, digestibility, microbial quality, preservation/extension of shelf life, nutritional, and health benefits as probiotics. Through fermentation, microbial cultures play a key role in food production. Fermentation utilizes the growth and metabolic activities of microorganisms to transform food materials (Terefe 2016). Food cultures are defined by the European Food & Fermentation Cultures Association (EFFCA) as live bacteria, yeasts, or filamentous fungi (moulds) used in food production where they are food ingredients. They usually have minimum viable cells count of 10^8^ CFU/g or ml for bacteria and yeasts, and 10^7^ CFU/g for filamentous fungi. Food culture preparations can be composed of one or several microorganism species with or without specific components (e.g. organic acids, minerals, vitamins) required for their survival, storage, or to facilitate their application in the final food.

Early Summer 2023, the European Commission issued a regulatory proposal on plants obtained by certain New Genomic Techniques (NGTs) and their food and feed. This proposal distinguishes some of these techniques from genetically modified organisms (GMOs) obtained by transgenesis and facilitates their placing on the market when they could also occur naturally or be produced by conventional breeding techniques. This proposal does not apply to deliberately released microorganisms, including food cultures. As confirmed by the EU Court of Justice ruling from 2018, most if not all microorganisms obtained through the use of NGT would still classify as GMO as defined by Directive 2001/18/EC.

The current EU regulatory framework, which analytically struggles to differentiate between products of classical techniques and of NGTs, places the EU food biotechnology industry at a disadvantage, especially compared to countries with more facilitating regulations. An adapted, more flexible regulatory framework for NGTs could significantly benefit the consumer, industry, and align with the EU policy ambitions.

This paper will demonstrate how NGTs applied to microorganisms could be beneficial to the sector, allowing food cultures to contribute to an innovative, safe, and sustainable food system, providing similar attempt for modernization of the legislation as for plants.

### The need to expand our toolbox beyond natural evolution of microbial genomes

Natural selection and laboratory evolution techniques have been used for many decades to improve microbial strains for use as food cultures, targeting traits like acid tolerance in yogurt and metabolite production for texture and aroma in food (Johansen 2018). *Streptococcus thermophilus*, a key organism in many dairy products, has undergone substantial genetic adaptation for milk-based growth, evidenced by major metabolic changes and gene transfer related to stress tolerance, exopolysaccharides production, and bacteriophage immunity (Hols et al. 2005, Eng et al. 2011).

Strain exposure to infection by bacteriophages leading to CRISPR (Clustered Regularly Interspaced Short Palindromic Repeats)-based immunization of the surviving strains against those phages is an even more active form of microbial genomic evolution (Barrangou et al. 2007). Barrangou et al. discovered that bacterial genomes pick up sequences over time from phages to which they were exposed, passing on these so-called CRISPR spacers to subsequent generations, then using these spacer sequences to recognize viruses that later invade their cells. The immediate practical application of this discovery was the avoidance of fermented food spoilage from phage infection. In addition, this discovery laid the foundation for the discovery of the CRISPR–Cas toolbox for genome editing (see NGTs available for deployment).

Beyond these, strain improvement techniques include natural competence processes like transformation and conjugation. Natural competence, controlled by a pheromone quorum-sensing system (Fontaine et al. 2010) allows for exchange of genetic material between related strains based on sufficient homology of the DNA being exchanged for recombination to occur, i.e. homologous recombination. Although this technique is more efficient than natural selection, it tends to require extensive screening after transformation to make sure that only the desired allele is exchanged between strains.

Techniques that rely on natural competence, even when performed in the laboratory, can lead to strain improvements. Such improvements are not classified as genetic modifications under Directive 2001/18/EC on the deliberate release of GMOs into the environment. This applies as long as these techniques do not involve the use of recombinant nucleic acid molecules or genetically modified organisms, except for those methods excluded by Annex IB of the Directive, like random mutagenesis.

Natural selection, adaptive evolution, and techniques based on natural competence are techniques of strain improvement that explore and deploy natural variation. Other techniques that do generate new genetic variation are also available, such as random mutagenesis. Random mutagenesis depends on UV irradiation or mutagenic chemicals to induce mutations in the bacterial genome, followed by screening for the desired phenotype and/or genotype. As this method generates new genetic variation, it is considered a method of genetic modification. However, it is listed in Annex IB of Directive 2001/18/EC as a method of genetic modification used before 2001 that is exempted from GMO requirements and labelling, as it does not involve the insertion of nucleic acids.

To date, food industries have screened and selected in the natural environment the proper microbial candidates to fulfill market demands. When necessary, natural strain selection strategies have successfully been applied to deliver on improvement of performance traits and safety standards (Derkx et al. 2014). However, the constantly increasing market requirements necessitate a broad diversification of foods due to specific dietary requirements, sustainability challenges, and higher safety standards, seeking effective solutions in a shorter time frame, all while maintaining high demands on product performance of the solutions provided by the food industries. Natural selection and classical improvement of microorganisms (e.g. random mutagenesis, hybridization, adaptive laboratory evolution) require laborious procedures and long time, particularly when a combination of multiple traits needs to be obtained from the product solution. To achieve the ambitious market demands, the industry needs to accelerate their innovation and broaden the scope of their application. To enable operators in the EU’s food sectors to meet this challenge, it will be necessary to equip them with modern tools.

Targeted mutagenesis is essentially a much more precise method of mutagenesis by which specific target nucleotides are deleted or altered using site-directed nuclease (SDN) enzymes and sophisticated repair mechanisms. Collectively, these SDN techniques are also referred to as NGTs, and their applications are discussed in the ‘Application examples of nontransgenic NGTs’ section. Nontransgenic NGTs applied to microorganisms are among the solutions to achieve these ambitious goals. These innovative techniques allow to introduce a precise, minimal DNA modification in microbial genomes, resulting in optimized products carrying features that could also be achieved by spontaneous natural genetic evolution, without insertion of heterologous material.

Due to the near identity in the genetic information between nontransgenic NGTs and classical improvement techniques (current and future), analytical methods may not be able to distinguish between the food cultures obtained by different techniques. This implies that the current technique-based GMO regulatory framework is not fit-for-purpose, and it is prone to noncompliance especially from imports from third countries, where certain NGT products benefit from a facilitating regulatory framework for marketing (e.g. USA, Canada, Australia, Israel and India, whereas in several other countries, a case-by-case approach is implemented). This places the EU food biotechnology industry at a competitive disadvantage.

The current EU regulatory framework discourages the development of microorganisms derived from NGTs and prevents consumers from benefitting from these products. NGTs bring opportunities to precisely alter the genetic material of microorganisms, allowing the rapid development of new microorganisms to face continuously evolving market needs and to satisfy the increasing variability of customers’ demands. Having an adapted and facilitating regulatory framework for NGTs on microorganisms would allow the industry to contribute efficiently to EU policy ambitions.

### NGTs available for deployment

In this section, we put the spotlight on the possibility to perform nontransgenic precision genome editing in microorganisms via NGTs, such as recombineering and CRISPR–Cas-mediated editing systems. These techniques have significantly expanded the genetic toolbox, allowing to precisely make genetic changes, such as point mutations in specific positions of bacterial genomes, without leaving any trace of exogenous DNA.

**Figure 1.**
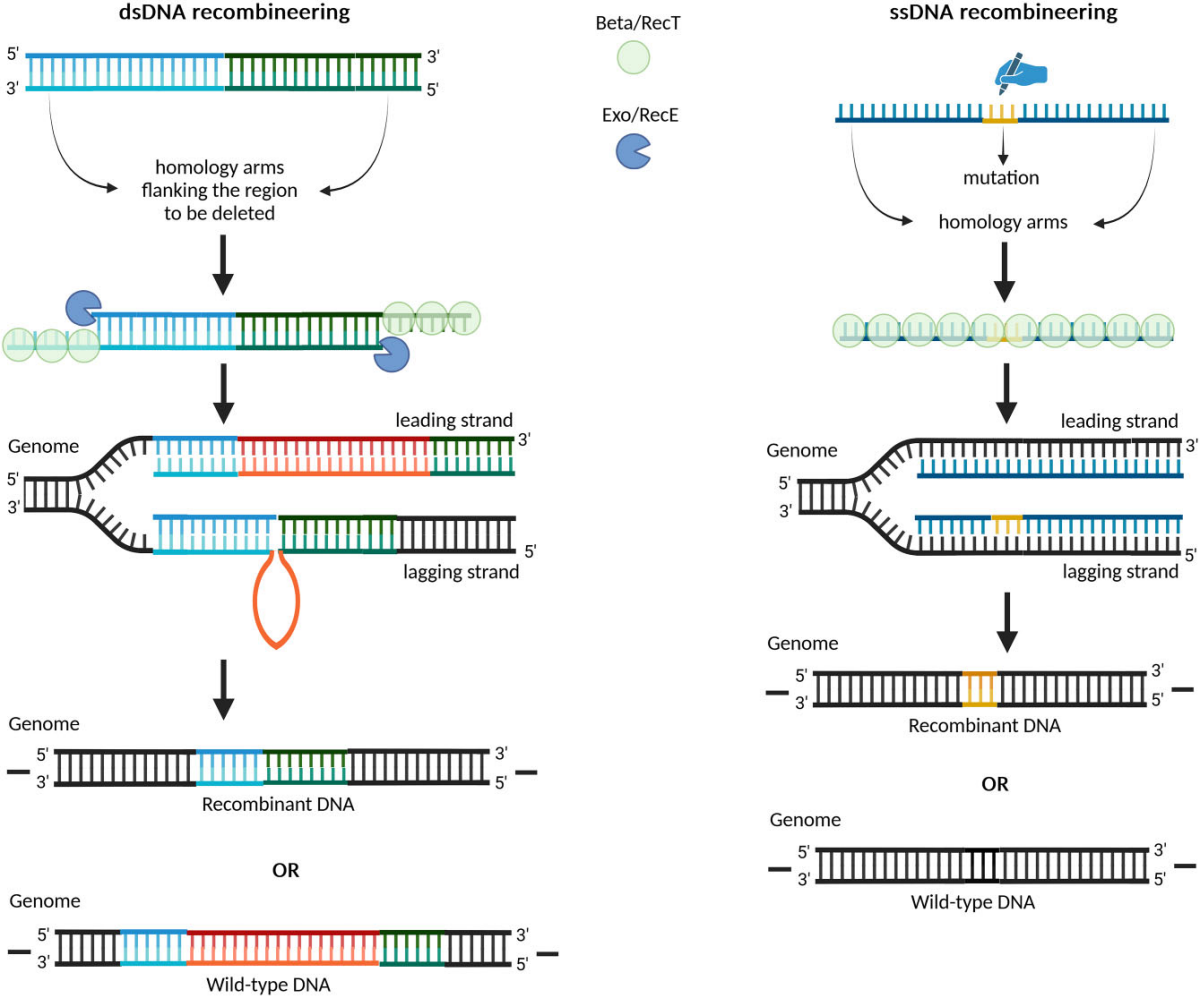
Precision genome editing via the recombineering technique. Recombineering DNA fragment can be double-stranded (dsDNA) or single-stranded (ssDNA). dsDNA molecules are degraded in the 5′ to 3′ direction by Exo/RecE exonucleases to generate ssDNA intermediates, which are protected from the degradation by Beta/RecT ssDNA-binding proteins. In addition, Beta/RecT promote the annealing of the ssDNA recombineering fragments to the complementary region on the target bacterial genome, thanks to the homology arms. Recombination occurring between the homology regions can result in wild-type or recombinant genotype, which needs to be checked by sequencing or polymerase chain reaction (in case of large editing). ssDNA molecules must pair with the lagging strand of DNA synthesis to optimize the recombineering efficiency. Created with Biorender.com.

Recombineering (recombination-mediated genetic engineering) is a genome editing system mediated by bacteriophage-encoded recombinase machineries (*λ*-Red and RecET). The technique allows for the direct integration of linear DNA fragments (either single-stranded or double-stranded), containing the desired mutation(s) flanked by homologous regions of the target DNA, into a microbial cell expressing the phage-derived recombination enzymes. These enzymes recombine the linear DNA at the target site, introducing the designed mutation into the microbial genome (Fig. 1).

The dsDNA recombineering approach has been exploited to generate strains carrying clean modifications, such as deletions, insertions, or replacements of large gene fragment, while ssDNA recombineering enables the engineering of point mutations in the genome without the use of selectable markers. The latter technique has been successfully established in several lactic acid bacteria (LAB) species, such as *Lactococcus lactis, Limosilactobacillus reuteri*, and *Lac. gasseri* (van Pijkeren and Britton 2012). Recombineering systems need to be optimized for each LAB strain to reach recombination frequencies sufficiently high for the selection of mutant cells without selective pressure. Interestingly, ssDNA recombineering has been successfully combined with CRISPR–Cas to simplify the selection of recombinant cells (van Pijkeren and Britton 2014).

CRISPR–Cas systems are widespread in bacteria and constitute their native adaptive immune system, providing defence against foreign nucleic acids, such as plasmids or DNA from viruses (Barangou et al. 2007, Bahya et al. 2011). The molecular mechanism of adaptive immunity is a phenomenon that was first experimentally confirmed in *S. thermophilus* by Danisco molecular biologists working with cheese and yoghurt cultures, who found that CRISPR sequences (bacteriophage DNA sequences inserted as DNA spacers separated by short palindromic repeats and grouped into clusters in intergenic regions) are naturally incorporated into the bacterial genome of phage-infected surviving strains and, together with Cas proteins, provide acquired resistance against viruses. The bacterial CRISPR spacers create a permanent record of phages against which the bacteria have mounted defences. Since the early production of cheese and yoghurt, people across the globe have been consuming these products made with starter cultures immune to phage infection due to this natural mechanism.

**Figure 2.**
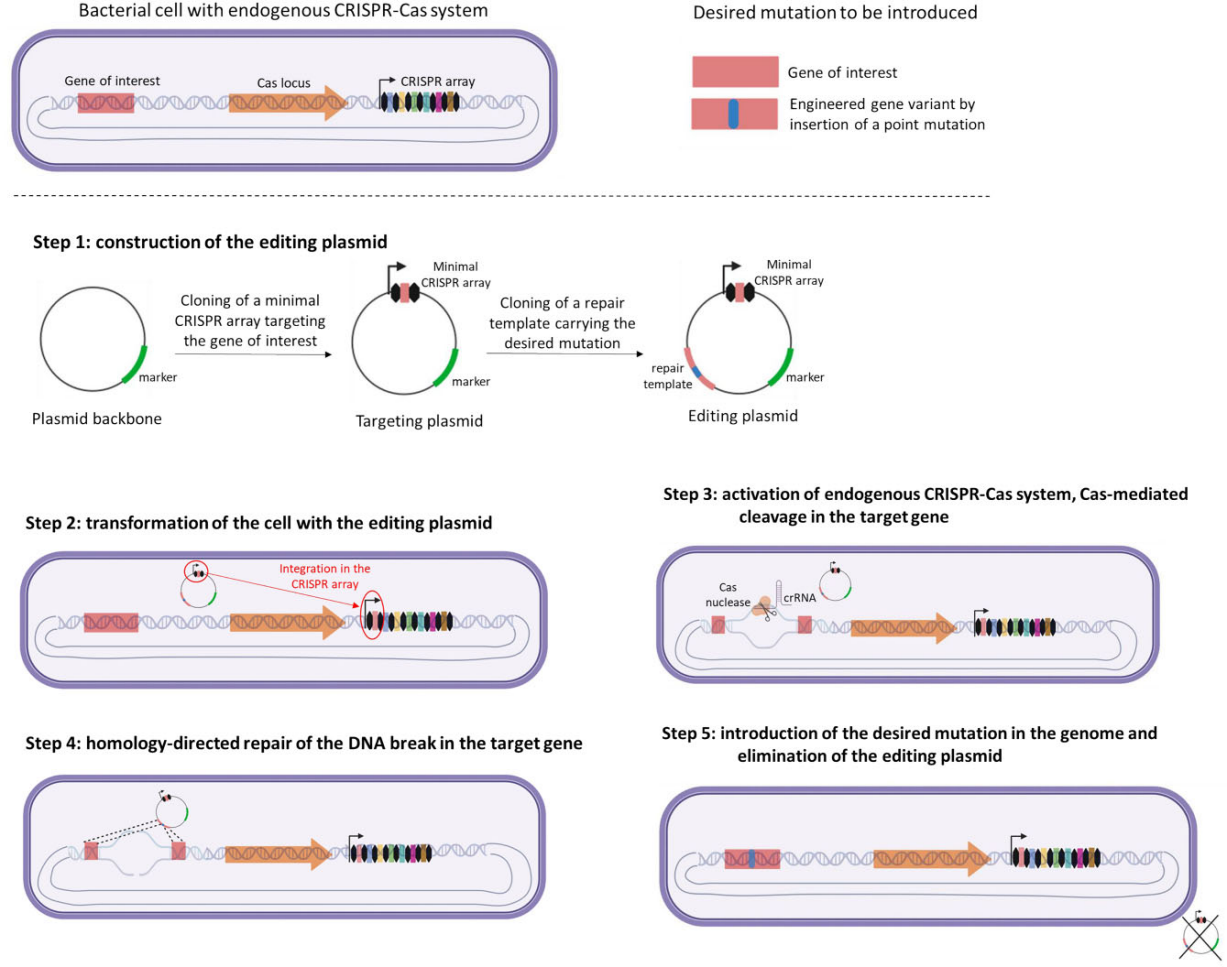
Workflow for reprogramming endogenous CRISPR–Cas system to perform precise genome editing. Created with Biorender.com.

The discovery of the role of CRISPR in immunity to phages ultimately led to the development of CRISPR–Cas-based genome editing by research groups at UC Berkeley and the Broad Institute at Harvard. CRISPR–Cas systems have since then been perfected and have become one of the most promising NGTs to accelerate precise and marker-free genetic improvement not only of prokaryotes but also of fungi, plants, and animals (Barrangou and Horvath 2017). For example, by utilizing the Cas9 enzyme and a guide RNA, researchers can target specific genomic loci and introduce precise point mutations (Komor et al. 2016). Endogenous CRISPR–Cas systems have been repurposed in LAB to introduce point mutations at a specific position of the genome in a precise, programmable, and efficient manner (Hidalgo-Cantabrana et al. 2019). Minimal CRISPR arrays with self-targeting DNA fragments could be engineered and delivered to the native host in combination with a mutated repair template, resulting in accurate genome editing (Fig. 2). This process simply exploits the natural ability of bacteria to overcome DNA damage by homology-directed repair with the provided DNA template (Fig. 2). The strain development is therefore achieved by the transient delivery of a plasmid that is lost from the cell after the editing (Fig. 2).

### Application examples of nontransgenic NGTs

**Figure 3.**
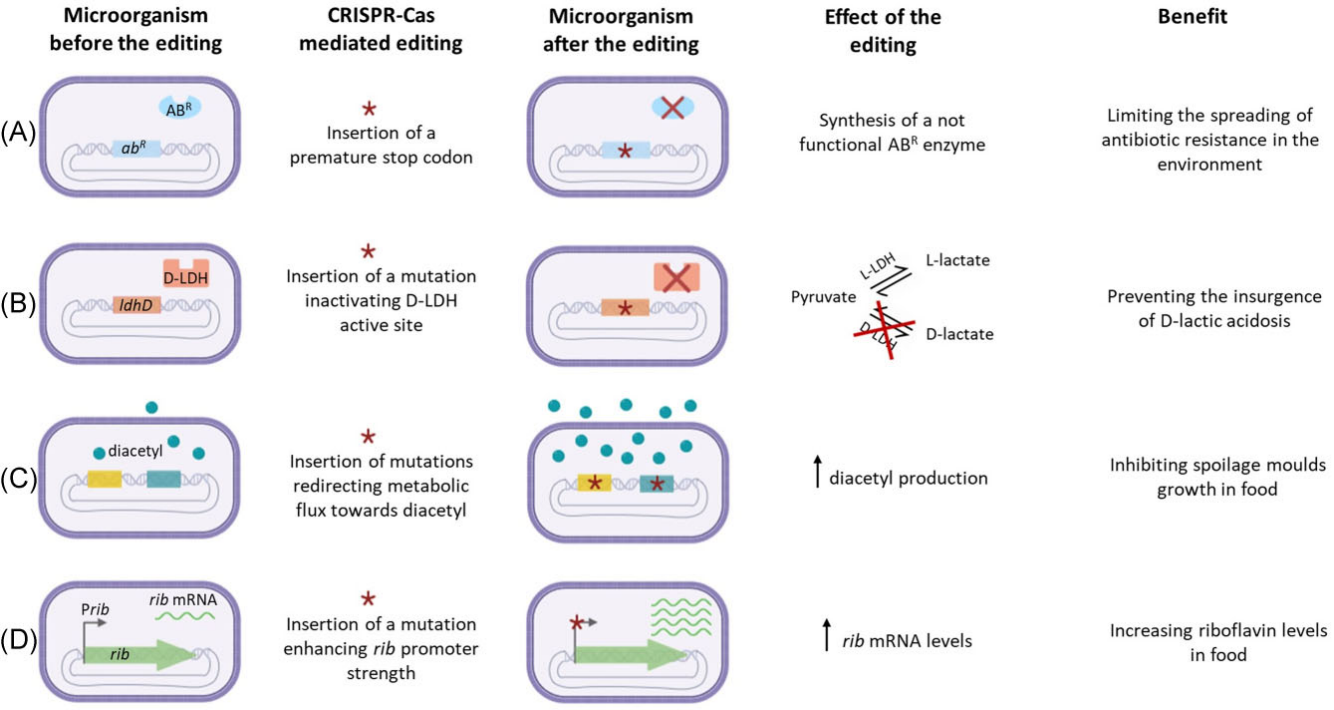
Nontransgenic NGT-mediated editing as a tool to improve food safety, sustainability, and quality. ab^R^, antibiotic resistance (gene); AB^R^, antibiotic resistance (enzyme); *ldhD*, d-lactate dehydrogenase (gene), d-LDH, d-lactate dehydrogenase (enzyme); and *rib*, riboflavin biosynthetic operon. Created with Biorender.com.

Research in food applications is looking for optimized safe food cultures not carrying undesirable traits (e.g. antibiotic resistance, production of d-lactic acid), and/or resulting in food products with improved nutritional value (e.g. higher vitamin content), increased organoleptic and rheological properties as well as extended shelf life. Microbial genetic improvement is the bottleneck in food culture developments, as it currently relies on lengthy natural genetic evolution strategies or on untargeted random mutagenesis. In this scenario, precise microbial genome editing by nontransgenic NGTs may be beneficial to food applications, developing strains that could enhance food safety, sustainability, and quality (Fig. 3).

Nontransgenic NGTs are nowadays exploited as research tools to gain insights into microbial pathways and understand the function of predicted genes, reducing the screening burden for the identification of improved strains after natural evolution or random mutagenesis campaigns (Derkx et al. 2014). These techniques have evolved from native microbial machineries and have the potentiality to design tailored food cultures having the desired properties with a targeted minimal DNA modification.

The endogenous CRISPR–Cas system was successfully reprogrammed in the probiotic *Bifidobacterium animalis* subsp. *lactis* to inactivate the *tetW* gene encoding resistance to tetracycline (Pan et al. 2022). Moreover, a targeted ssDNA recombineering machinery was developed and transiently expressed in a *Limosilactobacillus reuteri* candidate probiotic strain to create a vancomycin-sensitive variant (van Pijkeren and Britton 2012). Importantly, the introduction of a single amino acid change in the enzymatic target of the antibiotic, the D-ala D-ala ligase, was sufficient to lower the minimum inhibitory concentration for vancomycin from >256 to 1.5 µg/ml, well below the clinically relevant threshold. The inactivation of genes coding for antibiotic resistance in LAB transiting or colonizing the human gut would be pivotal to prevent the spread of such resistances to intestinal pathobionts, reducing the risk for the insurgence of multidrug-resistant bacteria (Fig. 3A). This has important implications not only for probiotics, but also for microorganisms used as starter cultures or cultures with protective effects. Nontransgenic NGTs may also be exploited to redirect LAB metabolic fluxes towards the production of high added-value molecules. Lactic acid is the primary product of LAB carbohydrate metabolism and is the most valuable product in the food industry, having also important applications in cosmetic, pharmaceutical, and agricultural sectors (Castillo Martinez et al. 2013). It exists in two enantiomeric forms, l- and d-lactic acid, which are produced by two different enzymes (l- or d-lactate dehydrogenase, respectively) and differs in the technological uses. Microbial fermentation can therefore result in the production of an optically pure isomer of lactic acid or a racemic mixture, according to the LAB species selected. Food industry prefers the (L+) isomer, since it is the only isomer that can be efficiently metabolized by humans. Moreover, elevated levels of d-lactic acid in the blood or other body tissues may lead to insurgence of the metabolic syndrome, d-lactic acidosis (Mack 2004). Highly optically pure d-lactic acid is synthesized by some LAB species, including *Lac. delbrueckii* and *Leuconostoc* sp. (Tashiro et al. 2011, Alexandri et al. 2022). Precision genome editing by nontransgenic NGTs could prevent d-lactic acid production by generating LAB variants encoding a nonfunctional d-lactate dehydrogenase (d-LDH; Fig 3B). A similar approach was already applied to develop a *Lacticaseibacillus paracasei* strain producing optically pure l-lactic acid after the interruption of *ldhD* gene via plasmid-based homologous recombination (Kuo et al. 2015).

Nontransgenic NGTs can also be used on yeasts to reduce the production of undesirable or toxic substances. Ethyl carbamate may be produced during fermentation of alcoholic beverages, from the reaction of ethanol with urea, raising health concerns in humans. CRISPR–Cas system has been used to disrupt the CAR1 gene encoding arginase of the well-known *Saccharomyces cerevisiae* yeast, to decrease the formation of this undesirable substance during ethanolic fermentation (Chin et al. 2021). A double deletion of CAR1 and GZF3 using the same technique allowed to reduce even more the content of ethyl carbamate (Jung et al. 2022).

The food industry is facing increasing pressure to create products with longer shelf life. This leads to a greater emphasis on utilizing natural microbial processes instead of relying on chemical additives. For instance, diacetyl is a by-product of fermentation by many LAB strains, responsible for the buttery flavour in many dairy products, which was recently shown to reduce food spoilage by its intrinsic antifungal activity (Shi and Maktabdar 2022). Redirecting metabolic fluxes towards the synthesis of higher amount of diacetyl represents an important strategy to contribute to the reduction of food waste. This could be achieved by inserting precise point mutations, through nontransgenic NGTs, in specific genes belonging to the glucose metabolic pathway (Fig. 3C). An engineered *Lac. lactis* strain overproducing diacetyl was developed by simultaneously inactivating the *aldB* gene, to prevent the enzymatic conversion of the diacetyl precursor *α*-acetolactate (*α*-AL) to acetoin, and overproducing the NADH oxidase enzyme, to redirect the metabolic flux away from lactic acid synthesis towards *α*-AL production (Hugenholtz et al. 2000).

Market demands on food quality have become increasingly critical, with raising consumers attention towards safe and tasty foods and beverages. Sensory characteristics of fermented products can be improved by acting precisely on microorganisms’ genes using nontransgenic NGTs. *Saccharomyces* FDC1 gene is involved in the production of 4-vinyl guaiacol, which gives a spicy and clove-like flavour in beers, so called phenolic character of beers. These phenolic off-flavours have been significantly reduced by induction of a loss-of-function mutation in the FDC1 gene of yeasts (Mertens et al. 2019). Improving sensory quality can also be a matter of increasing desirable flavour compounds. Yeast has a major role in winemaking when it comes to develop wine flavours. Production of some acetate esters by *Sac. cerevisiae* induces fruity and flower-like aromas (by producing isoamyl acetate and phenylethyl acetate). Overexpressing ATF1 could increase the synthesis of alcohol acetyltransferase I, which catalyzes the formation of acetate esters from acetyl coenzyme A (Vilela 2021). Precise combination of genes overexpression and/or attenuation achieved via the use of nontransgenic NGTs would allow inhibiting or developing several specific flavours in fermented foods and beverages.

Increasing food nutritional value should also be addressed to relieve the pressure on food systems, considering the high nutritional requirements of the growing population. Several microorganisms are capable of producing substances with a high nutritional profile for humans, including essential amino acids, vitamins, minerals, and antioxidants (Graham and Ledesma-Amaro 2023). Group B vitamins are normally assimilated with the diet and play important roles in human cells metabolism. Despite its presence in foods, deficiencies in vitamin B2, known also as riboflavin, are very common in both developing and industrialized countries and are associated with several pathologies (Thakur et al. 2016). Riboflavin production has been observed in several LAB used in food culture formulations, and it is related to the presence of an intact riboflavin biosynthetic (*rib*) operon (Thakur et al. 2016). Introducing point mutations in the promoter region of the *rib* operon via nontransgenic NGTs would help boosting riboflavin production in food cultures (Fig. 3D). In fact, riboflavin production has been enhanced in a *Lac. lactis* subsp. *cremoris* strain when all four biosynthetic genes were simultaneously overexpressed in a plasmid (Burgess et al. 2004).

## Conclusion

The possibility to use nontransgenic NGTs would guarantee an improved scenario for food industries, which are part of the solution to achieve the ambitious targets set by the European ‘Green Deal’ and ‘Farm to Fork’ policies in a meaningful time frame widening their opportunities to generate innovative, safe, and more sustainable products for European consumers. This use is hindered by the current process-centric GM regulatory framework that is not fit for this purpose, having been established before the era of NGTs. The risk assessment and management of the final product/microorganism should be based on actual genome properties, as it is the case for those than can be achieved spontaneously in nature or by conventional breeding techniques, and not on the technique itself. This would also ensure the adaptability of the framework to new future techniques achieving the same type of products, thereby precluding any future lag between the evolution of the science and market access of the concerned products, ensuring European consumers can quickly benefit from innovative and safe products.

The revision of the EU regulatory framework is necessary as the EU market is missing opportunities for innovation, which is crucial to achieve the ambitious targets set by the European ‘Green Deal’ and ‘Farm to Fork’ policies in a meaningful timeframe. In other regions of the world, pragmatic regulatory frameworks are already in place. As a result, certain products are exempted from requirements of GM (and equivalent) regulatory frameworks in the USA, Canada, Australia, Israel, and India, whereas in several other countries, a case-by-case approach is implemented. EU is currently prone to noncompliance of imports from these third countries, where some NGTs products are already being marketed, as analytical methods capable of identifying nontransgenic products obtained by NGTs are missing.

As a first step towards a product-centric approach, microorganisms obtained by nontransgenic NGTs should be exempted from the obligations imposed by the EU GM regulatory framework for deliberate release, as is already the case for those obtained by conventional mutagenesis, where ‘nontransgenic’ events are identical to those spontaneously happening in nature.
